# Factors influencing health information acquisition behavior from a social capital theory perspective: a study of Zhihu users

**DOI:** 10.3389/fpubh.2025.1501879

**Published:** 2025-04-10

**Authors:** Juncheng Jia, Zemenghong Bao, Haochen Wang, Yuqun Cao, Luhan Yu, Kun Lv

**Affiliations:** ^1^Business School, Ningbo University, Ningbo, China; ^2^School of Cultural Heritage and Information Management, Shanghai University, Shanghai, China; ^3^School of Electrical and Computer Science, Ningbo University, Ningbo, China; ^4^Merchants’ Guild Economics and Cultural Intelligent Computing Laboratory, Ningbo University, Ningbo, China

**Keywords:** social capital, health inequality, fsQCA, health information acquisition behavior, influencing factors

## Abstract

The rapid development and widespread use of Internet technology have facilitated access to health information for the general public. However, the behavior of acquiring health information is influenced by multiple factors, resulting in differences and even inequalities. This paper aims to explore the influencing factors of user health information acquisition behavior, find feasible ways to optimize such behavior and maximize the utility of health information for users. By utilizing the Zhihu Q&A platform to obtain user health information and integrating social capital theory, the study identifies the influencing factors of user health information acquisition behavior. It utilizes fuzzy set qualitative comparative analysis to examine these factors as antecedent variables, with the dissemination heat of health information as the outcome variable. The study reveals that the structure, relationships, and cognitive dimensions of social capital significantly impact user health information acquisition behavior. The configuration path for user health information acquisition behavior generated by the fsQCA 3.0 software provides recommendations to maximize the utility of health information.

## 1 Introduction

For a long time, objective health disparities have existed among different groups (1). The development of mobile Internet technology has not only changed the operation mode of the medical and health industry but also profoundly affected the public’s access to health knowledge (2). The rapid development of information sharing, online medical care, virtual social interaction, and other aspects has turned the internet into an expanded space for obtaining health promotion resources (3). Online medical health platforms in China such as Dingxiang Doctor, and Medication Assistant, health management apps such as Keep, and Mint Health, and health content dissemination platforms such as TikTok and Zhihu, rely on the internet to continuously grow and expand. The diversification of user access to health information has allowed knowledge that was once only accessible through doctors, books, and magazines to reach the general public through fragmented reading patterns, making the Internet the primary source for seeking health information (4).

However, the diversification of health information acquisition behavior methods has also brought about differentiation. Even in the same network environment, different users have varying abilities to access health information. This has led to issues of information inequality (5), particularly in health research, where information inequality often stems from differences in social capital across different groups. Social capital generally refers to the ability of individuals or groups to access resources, support, and information within a social network, and it is closely related to an individual’s health status (6). For example, individuals with stronger social capital are often able to acquire health information, medical resources, and social support more effectively, which in turn contributes to better health outcomes. In contrast, groups with weaker social capital face difficulties in accessing health resources and information due to a lack of effective social support and information channels, leading to poorer health conditions and exacerbating health inequality (7). Therefore, social capital is a critical theory in the study of health inequality. It helps explain the differences in health information acquisition across different social groups. Based on this, this paper applies social capital theory to explore the factors influencing users’ health information acquisition.

## 2 Literature review

### 2.1 Definition of information inequality

Information inequality refers to the diverse information gaps formed at various levels of subjects in information and communication technology access and usage, as well as in the development and utilization of information resources (8). With the internet being fully integrated into social life, the mode of information flow and interaction between individuals has gradually changed the original social relationships and structures (9). The mastery of information and the ability to access information have become important considerations (10). However, due to differences in resource endowments, individuals have unequal abilities to access information (11). Research on this issue has been ongoing for a long time.

### 2.2 The concept of the digital divide

The concept of the digital divide was first proposed by the National Information Management Agency of the United States, initially exploring the rudiments of information inequality. It was believed that there was a gap between those who had access to new technology and those who did not, specifically in terms of the ownership of personal computers, broadband network connectivity, and other aspects (12, 13). Those who possessed technological devices were able to access effective information resources through the internet (14), creating a divide and causing information inequality compared to those who could not afford it (15). However, studies on the digital divide often used a simple binary division of information owners and the impoverished, assuming that users in different locations and with different demographic characteristics formed the divide (16). It mainly focused on the unequal opportunities for technology usage but paid less attention to the social reasons behind it (17). Up to now, the digital divide has been rarely discussed in the context of social inequality theory, other types of inequality, or general human inequality concepts (18).

### 2.3 Comparison between information inequality and the digital divide

In contrast, information inequality is a more comprehensive concept that transcends the limitations of the digital divide (19). Information inequality not only considers demographic variables, but also, due to its close association with offline social inequality, it also concerns the performance of digital technology in influencing social welfare, family, political participation, and social support (20). This viewpoint believes that the unequal distribution of status and power in social networks can lead to unequal participation in other areas of society (21, 22), just as in the field of health, where users’ unequal social capital leads to health inequality. Research on digital inequality often starts from a social stratification perspective (23), and the views of Bourdieu have received significant attention, holding that the concept of social capital can well explain the formation mechanism of digital inequality, where individuals with similar social spatial positions compete for resources and form insurmountable barriers (24).

### 2.4 Social capital theory and its relationship with health information acquisition behavior

Social capital refers to the sum of resources embedded in social networks that can be acquired and utilized (25). Initially proposed by the scholar Bourdieu, the amount of social capital possessed by a subject depends on the scale of the relationship network that they can effectively mobilize. Social capital has evolved from a marginal concept into a multidimensional concept, with differences in its concept and measurement indicators in different research contexts. Among them, the most widely applied classification of social capital is divided into three dimensions: structural capital, relational capital, and cognitive capital (26). Structural capital, which arises from objective factors such as connections, rules, and procedures among individuals, describes the frequency and duration of interaction between network members, emphasizing social interaction and connectivity within networks. Research indicates that the closer the connections between members in virtual communities, the more effective the sharing of knowledge (27). In the context of the internet, social interaction and connectivity refer to interactions such as questioning, answering, and discussing among users in online virtual communities. Users within strong network connections exhibit a stronger willingness to communicate and share, facilitating the transmission of information through frequent communication and making it easier to access relevant health information (28). Relational capital refers to the interpersonal relationships developed through communication and interaction between individuals, including trust, reciprocity, and identification. When individuals establish high levels of trust and reciprocal relationships within their social networks, they are more likely to obtain reliable health information from others in their network. For example, the presence of trust encourages individuals to share health experiences and advice, thereby improving the efficiency and quality of information transmission. Furthermore, a strong social support network can provide emotional support and psychological comfort, further enhancing individuals’ ability to acquire and understand health information. Cognitive capital refers to the common perceptions, values, and language used by individuals regarding things, while cognitive social capital arises from subjective factors such as individual consciousness, values, and attitudes (29, 30). When individuals can find health concepts and consistent health beliefs they identify with within their social networks, they are more likely to acquire and effectively use health information. Furthermore, the cognitive dimension also influences whether individuals can extract useful information for themselves, which is particularly important in the process of understanding health education and health behavior change. The various dimensions of social capital, through the connection and exchange of resources, contribute to the creation, exchange, and transmission of knowledge (31).

### 2.5 Health inequality under the fundamental cause theory

The study of health inequality often focuses on the field of sociology, with a particular emphasis on fundamental cause theory. This theory, similar to social capital theory, posits that socioeconomic status is the fundamental cause of health disparities and health inequality (32). It contends that individuals of higher social and economic standing possess a significant amount of resources, which can reduce the likelihood of illness and death, resulting in better overall health (33). The theory asserts that as long as there is inequality in the distribution of resources, health inequality will persist. Overall, fundamental cause theory contextualizes health research within a broader framework of social inequality, shifting the focus toward social stratification and inequality (34).

### 2.6 Research on health inequality under social capital theory

Both studies on digital inequality and health inequality recognize resources as a critical factor in perpetuating inequality. Social stratification theory maintains that the distribution of social resources is uneven: individuals of higher status have greater access to resources (35). The advantaged class is more knowledgeable about where to seek health information and better equipped to understand it (36). However, few studies have incorporated the ability to access health information into their research. The field of information science often studies user information behavior based on comprehensive information retrieval models but rarely includes the phenomenon of health inequality in its scope (37). Therefore, based on social capital theory, this paper seeks to explore the factors influencing user access to health information and employs fuzzy set qualitative comparative analysis to conduct configurational research. The aim is to identify feasible approaches to optimize user access to health information and maximize the utility of such information, ultimately alleviating health inequality.

## 3 Research design

### 3.1 Methodology and procedures

Qualitative Comparative Analysis (QCA) is a configurational analysis based on Boolean algebra and set theory. Its purpose is to explain the configurational phenomena of interdependent conditional factors and their resulting outcomes in practice (38). This method, rooted in a configurational perspective, explores the multiple concurrent causal relationships among variables, where various combinations of variables collectively lead to the same outcome (39).

**Figure 1:**
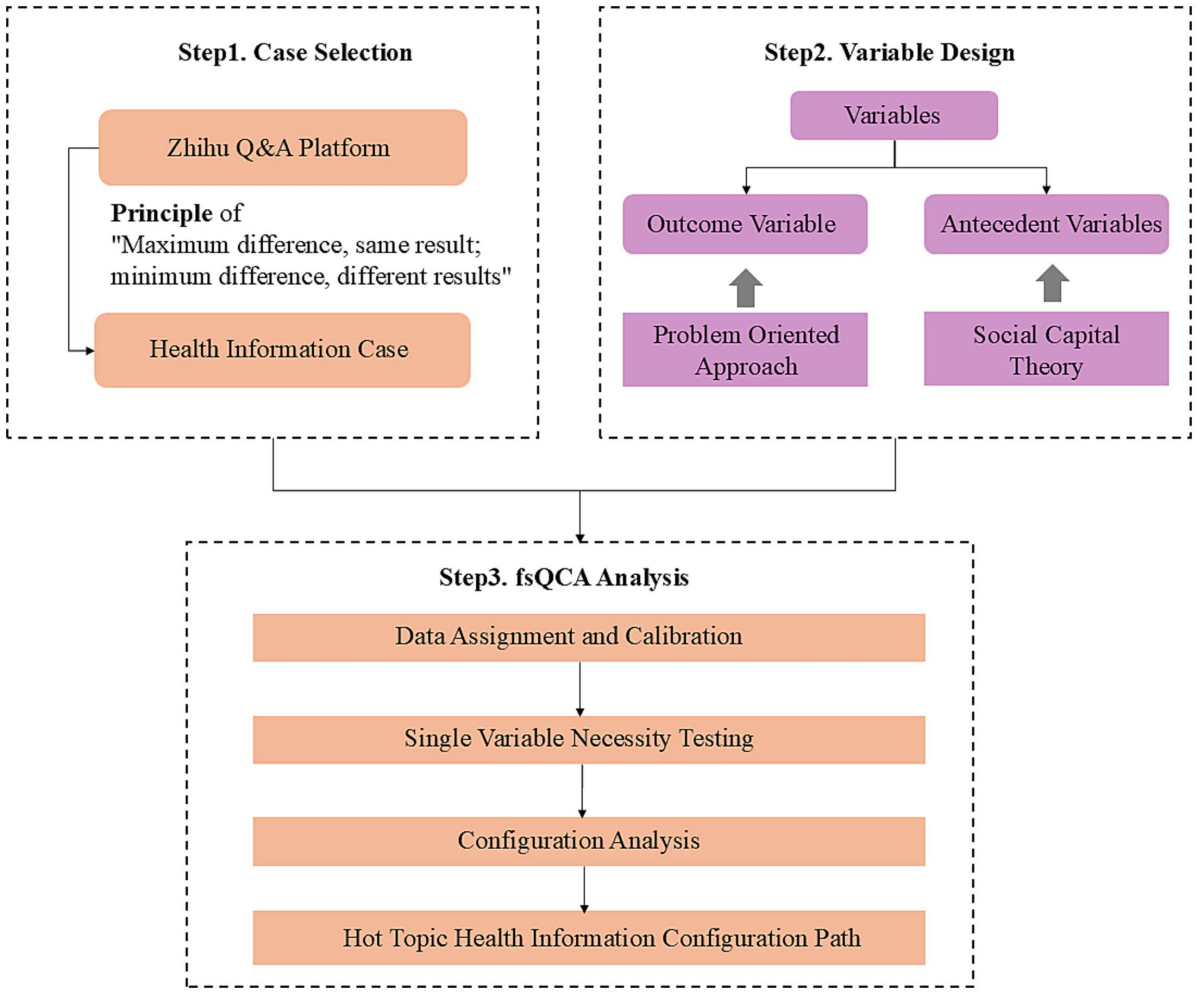
Research framework diagram.

Fuzzy Set Qualitative Comparative Analysis (fsQCA) is a type of qualitative comparative analysis method that allows for the scaling of set scores (40). In line with the focus and objectives of this study, the following research steps are formulated: (1) using the principle of “maximum difference, same result; minimum difference, different result,” (41) select health information from social Q&A platforms as the research case; (2) design antecedent and outcome variables based on case characteristics and social capital theory; (3) calibrate data based on variable data and case characteristics; (4) conduct necessary tests for single variable inclusion to preliminarily identify the core conditions leading to the appearance of the outcome variable; and (5) minimize configurations to output the final results. The specific research and analysis framework is illustrated in Figure 1.

### 3.2 Case selection

Health information, as defined in the 2014 General Medicine and Community Health vocabulary, encompasses all knowledge, techniques, skills, concepts, and behaviors related to human health produced, transmitted, and shared by both parties in the health communication process (42). This study unfolds an exploration using Zhihu (the Chinese equivalent of Quora) as an example, selecting health-related topics as the sample. To ensure the accuracy and effectiveness of the results, the principle of “maximum difference, same result; minimum difference, different result” is employed to select topics with comprehensive user information (43). In this study, the principle of “maximum difference, same result” is applied based on the health information dissemination heat (such as the total number of comments, likes, etc.), ensuring that, within the same outcome variable, the selected cases exhibit significant differences across other variables (such as region, industry, education level, etc.). This approach helps ensure the diversity and generalizability of the study. On the other hand, for cases with substantial differences in health information heat, the study employs the principle of “minimum difference, different results,” selecting cases where differences across other variables are minimal. Case selection based on this principle helps eliminate those cases whose extreme differences would render the results less generalizable, thereby ensuring that the selected cases possess high representativeness for the configurational analysis.

The specific cases and basic information are presented in Table 1.

**Table 1:** fsQCA case.

Number	Case name	Number	Case name
1	Is it true that all beverages taste better when served cold?	16	What is your view on the article published in “Science” that suggests middle-aged weight gain is not due to a slowing metabolism?
2	Is it true that all beverages taste better when served cold? (2)	17	What is your view on the article published in “Science” that suggests middle-aged weight gain is not due to a slowing metabolism? (2)
3	What are the methods for sobering up?	18	What are the benefits of jogging five kilometers every day?
4	What are the methods for sobering up? (2)	19	What are the benefits of jogging five kilometers every day? (2)
5	A woman suffered from facial paralysis after riding a bicycle with wet hair.	20	Why is milk still widely promoted in China when most people are lactose intolerant?
6	A woman suffered from facial paralysis after riding a bicycle with wet hair. (2)	21	Why is milk still widely promoted in China when most people are lactose intolerant? (2)
7	What fruits are best for people with diabetes to consume?	22	What eating habits do thin people have?
8	Are there any benefits to being overweight?	23	What are your small habits for looking beautiful?
9	Are there any benefits to being overweight? (2)	24	What does the real life of a gout patient look like?
10	A 16-year-old boy died after choking on a pearl milk tea, his family claims it was due to sudden cardiac arrest. How should one handle such situations promptly?	25	Why am I still tired after getting at least 8 h of sleep every day? How can I adjust my body to be energetic?
11	How can men maintain their health daily?	26	How can one determine which vitamin they are deficient in?
12	Could you recommend some toothpaste that truly has whitening effects on teeth?	27	Why does a person who brushes their teeth diligently have bad breath?
13	Could you recommend some toothpaste that truly has whitening effects on teeth? (2)	28	How can office workers protect their shoulders and necks from strain?
14	Do foreign girls drink ice water and their mothers do not tell them it’s bad for their health and may cause cold in the womb?	29	How can office workers protect their shoulders and necks from strain? (2)
15	What signs on your body indicate that you are aging?	30	How can one control their uric acid levels to maintain normalcy?

### 3.3 Variable inclusion

Zhang Ming et al. proposed five main methods for determining conditions in QCA research: the problem-oriented approach, the research framework approach, the theoretical perspective approach, the literature induction approach, and the phenomenon summarization approach (44). Among these, the theoretical perspective approach matches corresponding conditions based on different theories or explanatory perspectives. In this study, the theoretical perspective approach is employed to determine the antecedent variables, while the problem-oriented approach is used to determine the outcome variables. The theoretical framework selected for this study is the social capital theory.

As social Q&A platforms serve as a primary source of knowledge acquisition behavior in the Internet era, users’ engagement impacts their access to health information (45). Therefore, this paper applies the social capital theory to measure the relationship between social capital and the health information-seeking behavior of social Q&A platform users from three dimensions: relational, cognitive, and structural. Zhihu users’ social capital is categorized into three types (see Table 2) based on this framework.

**Table 2:** Social capital classification of Zhihu users.

Category	Structural	Relational	Cognitive
Offline	Locale, industry	Trust, mutual benefit, identification	Education occupation
Online	View count, comment count	Follower count Following count	Like count

For the structural social capital variables, structural social capital emphasizes an individual’s position within a social network and the structure of that network. Among the variables selected in this study, offline factors such as region and industry reflect the distribution of an individual’s resources, which influences the information and resources they can access. Online behaviors, such as the number of views and comments, reflect the frequency of user interactions on the platform and the activity level of their social network. Therefore, these variables effectively measure an individual’s structural social capital.

For the relational social capital variables, relational social capital primarily focuses on the quality of social relationships between individuals, especially the role of trust, reciprocity, and recognition in facilitating information exchange. Among the variables selected in this study, trust and reciprocity help enhance the dissemination and credibility of health information, while recognition strengthens an individual’s sense of participation and belonging within the social network. In terms of online behaviors on the platform, the number of followers and the number of people followed reflect the accumulation of social capital, indicating the individual’s ability to access more information and exert greater influence.

For the cognitive social capital variables, cognitive social capital concerns an individual’s knowledge background, cognitive ability, and values. Among the selected variables in this study, education level and occupation reflect the individual’s knowledge background and information processing capabilities, influencing their understanding and application of health information. In terms of online behaviors, the number of likes received reflects the user’s contribution to and recognition of health information, indicating strong social cognitive capital on the platform.

### 3.4 Data assignment and calibration

By the requirements of fsQCA analysis, the antecedent and outcome variables in the sample need to be calibrated before configurational analysis, transforming them into membership values on the (0,1) set. This is typically achieved through three-value assignment, four-value assignment, six-value assignment, or continuous assignment methods (46). Among these methods, the four-value assignment approach strikes an optimal balance between precision and simplicity. In contrast, the three-value method, while simplifying the categorization of variables, proves overly coarse, failing to capture subtle differences between variables. On the other hand, the six-value assignment method, with its inclusion of additional intermediate values, increases analytical complexity, thereby hindering model parsimony. Furthermore, within the four-value assignment framework, the values of 0 and 1 signify states of complete “non-membership” and “full membership,” respectively, enabling a clear delineation of extreme conditions. Meanwhile, the intermediate values of 0.33 and 0.67 denote states of “low membership” and “high membership,” respectively, effectively representing variables within ambiguous boundaries and facilitating a more nuanced depiction of data variability. Given that numerous variables, such as users’ social capital and the frequency of information acquisition, exhibit a degree of fuzziness in the context of health information retrieval, the four-value assignment method proves superior in addressing this ambiguity and enhancing the model’s explanatory power. Based on the four-value method, the specific assignments for each antecedent variable are outlined in Table 3.

**Table 3:** Antecedent variable assignment table.

Variable name	Target set	Assignment	Specific meaning
Structural social capital	User region	1	Economically developed regions
		0.67	Regions with moderate economic development
		0.33	Economically underdeveloped regions
		0	Impoverished areas/no data
	User industry	1	Industries related to health information
		0.67	Industries somewhat related to health information
		0.33	Related but does not directly/indirectly impact
		0	Unrelated industries
Relational social capital	Interacting users	1	Users with extremely high interaction frequency
		0.67	Users with high interaction frequency
		0.33	Users with moderate interaction frequency
		0	Users with low interaction frequency
Cognitive social capital	User education level	1	High-level university/health-related majors
		0.67	High-level university/non-related majors
		0.33	Average university
		0	Not applicable/no data
	Number of likes received	1	Extremely high number of likes received
		0.67	High number of likes received
		0.33	Moderate number of likes received
		0	Low number of likes received

By the problem-oriented approach (47), this study selects the number of question-answer comments and endorsements as outcome variables (The Zhihu research data in this study is up to June 2024). Direct calibration method is employed for encoding, with 3,000 set as the threshold for complete membership (48), assigning a membership score of 0.95; 200 as the threshold for complete non-membership, with a membership score of 0.05; and 600 as the crossover point, from which the membership score is calculated (The direct calibration method process is provided in Table A1 of Appendix A).

The 30 topic cases in Table A1 of Appendix A are coded and assigned values based on the characteristic properties of the antecedent and outcome variables, resulting in a measurement of the antecedent and outcome variables (The measurement results are shown in Table A2 of Appendix A).

## 4 Data analysis

### 4.1 Single condition necessity analysis

Consistency and coverage calculations for a single independent variable are conducted to determine whether the independent variable is a sufficient and necessary condition for the dependent variable. Generally, when the consistency is ≥0.9, the independent variable can be considered a sufficient and necessary condition for the dependent variable. The calculation formula is shown in the equation:


$$\mathrm{Consistancy}\left(Y_{i}\leq X_{i}\right)=\sum \frac{\min\left(X_{i},Y_{i}\right)}{\sum \left(Y_{i}\right)}$$

The data is imported into the fsQCA3.0 software for computation. Adding a “~” in front of a variable indicates the non-existence of that variable, and the results are presented in Table 4.

**Table 4:** Table for the necessity of single conditions.

Antecedent variables	Result variables	
	Popularity	~Popularity
Region	0.883942	0.847853
~Region	0.207299	0.228834
Industry	0.522628	0.465031
~Industry	0.531387	0.580368
Interactive users	0.691241	0.692638
~Interactive users	0.435766	0.414110
Education level	0.448175	0.466258
~Education level	0.628467	0.598160
Number of likes received	0.605109	0.463190
~Number of likes received	0.506569	0.630675

“~” indicates the non-existence of the variable.

From the results, it is evident that the consistency values of all the antecedent variables did not reach 0.9, indicating a preliminary assessment of the weak explanatory power of the variables in influencing users’ health information acquisition behavior. No definitive factors or essential conditions influencing this behavior were identified. Consequently, this study will further investigate the configurational effects among the antecedent variables.

### 4.2 Configuration analysis

A truth table was constructed using fsQCA3.0 software, and the table was then edited. The case threshold was set at 1 and the consistency threshold at 0.8. Configuration pathways with fewer cases were removed to simplify the analysis, ultimately yielding three types of solutions: complex solutions, intermediate solutions, and simple solutions. This study only presents the intermediate solution, as detailed in Table 5.

**Table 5:** Intermediate solution results.

Configuration	Raw coverage	Unique coverage	Consistency
~Region * ~ Industry * Education Level	0.0343066	0.0102189	0.701493
Region * ~ Industry * ~ Interactive Users * ~ Education Level	0.3	0.250365	0.727434
Region * Industry * Interactive Users * Number of Likes Received	0.409489	0.359854	0.672662
~Region * Industry * Interactive Users * ~ Education Level * ~ Number of Likes Received	0.0751825	0.0510948	0.774436
Solution Coverage	0.745255		
Solution Consistency	0.695978		

“~” indicates the non-existence of the variable.

“*“*” indicates the intersection relationship between variables, which is used to connect variables.

In Table 5, “*” serves as the connector symbol indicating an intersection between variables, while “~” denotes the absence of a variable. “Raw coverage” represents the proportion of the impact of a conditional combination formed by antecedent variables on the solution, measuring the membership degree of each component in the combination on the solution’s impact. “Unique coverage” illustrates the proportion of the solution’s interpreted membership degree of a specific conditional combination formed by antecedent variables, reflecting the interpretive ability and importance of a particular combination on the solution, generally serving as the primary basis for analysis. “Consistency” is used to determine whether a specific combination of antecedent variables is a sufficient condition for the solution. “Solution coverage” denotes the overall coverage of all combinations of antecedent variables for the solution, while “solution consistency” represents the consistency indicator for all conditional combinations.

In practical analysis, due to the superior interpretive and adaptive qualities of the intermediate solution for the issue under study, which surpasses both the complex and simple solutions, the majority of studies employ the intermediate solution for analysis. Qualitative comparative analysis theory posits that if a condition appears in both the simple and intermediate solutions, it is defined as a core condition, whereas if it only appears in the intermediate solution, it is considered an auxiliary condition (49). Integrated with the simple and intermediate solutions, this study ultimately establishes four antecedent variable conditional combinations influencing the outcome variables:

1. H1: ~*Region* * ~ *Industry* * *Education Level*;
2. H2: *Region* * ~ *Industry* * ~ *Interactive Users* * ~ *Education Level*;
3. H3: *Region* * *Industry* * *Interactive Users* * *Number of Likes Received*;
4. H4: ~*Region* * *Industry* * *Interactive Users* * ~ *Education Level* * ~ *Number of Likes Received*.

These variable combinations are presented as shown in Table 6.

**Table 6:** The path of hotspot health information configuration.

Antecedent variables	Hotspot health information configuration			
	H1	H2	H3	H4
Region	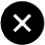	⊕	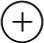	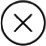
Industry	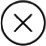	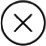	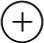	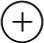
Interactive users		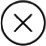	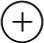	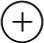
Education level	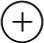	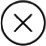		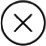
Number of likes received			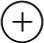	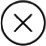
Consistency	0.701493	0.727434	0.672662	0.774436
Raw coverage	0.0343066	0.3	0.409489	0.0751825
Unique coverage	0.0102189	0.250365	0.359854	0.0510948
Solution consistency	0.695978			
Solution coverage	0.745255			

Table 6, ⊕ indicates the presence of a core condition, 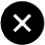 indicates the absence of a core condition, ⊕ denotes the presence of an auxiliary condition, and 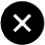 signifies the absence of an auxiliary condition. Blank spaces represent conditions that may or may not exist. The overall consistency of the solution is 0.695978, indicating that approximately 69.6% of the cases analyzed can be explained by the selected combination of conditions, suggesting a degree of reliability but not complete biaslessness. Consequently, it can be inferred that while the study has identified relatively significant patterns across the four pathways of hot topic formation, a proportion of unexplained variance remains, necessitating further exploration of other potential factors. Additionally, the overall coverage of the solution is 0.745255, indicating that these four configurational pathways can account for about 74.5% of the reasons for health information acquisition. This high coverage value suggests that the formation of most hot topics can be reasonably explained by these four pathways, yet it also hints at the presence of some external variables or complex interactive effects that were not included in the analysis of this study.

## 5 Analysis and discussion

### 5.1 Analysis of the impact of single antecedent variables

Derived from social capital theory, the factors influencing users’ acquisition behavior of health information can be categorized into three types of social capital: structural, relational, and cognitive. This study, utilizing fuzzy and qualitative comparative analysis, employs the aforementioned categories of social capital—*Region*, *Industry*, *Interactive Users*, *Education Level*, and *Number of Likes Received*—as antecedent variables, with the health information popularity (sum of comments and likes) as the outcome variable. The findings reveal that while all three types of social capital—structural, relational, and cognitive—impact users’ acquisition behavior of health information, the influence of single factors is relatively weak and not a sufficient condition for the formation of hot topics. Subsequent sections will elucidate the extent of the impact of each antecedent variable on the formation of hot topics based on the analysis of single-condition necessity.

In particular, the impact of the region within the structural social capital on users’ acquisition behavior of health information is significant, with a consistency of 0.883942. Offline social capital such as region can influence users’ online information behavior, as users in economically developed regions have access to more convenient technological devices and technological education. As a result, users can alter their structural social capital through migration and other means to facilitate their acquisition behavior of health information. Relational social capital can also have a certain impact on users’ acquisition behavior of health information, with a consistency of 0.691241. The more followers and followers a user has, the greater the likelihood of encountering health information. Therefore, users can accumulate their relational social capital by increasing their internet usage frequency and networking frequency to broaden their channels for acquiring health information. However, the impact of offline social capital education within cognitive social capital is not particularly pronounced, with a consistency of only 0.448175, and the impact of online social capital likes count is also relatively minor, with a consistency of 0.605109. Education and the user’s knowledge literacy alone do not play a decisive role in the acquisition behavior of health information. Therefore, when offline cognitive capital is difficult to change, users can improve their online cognitive social capital through their efforts, such as continuing their education, to improve their environment for acquiring health information.

### 5.2 Configuration path analysis

**Figure 2:**
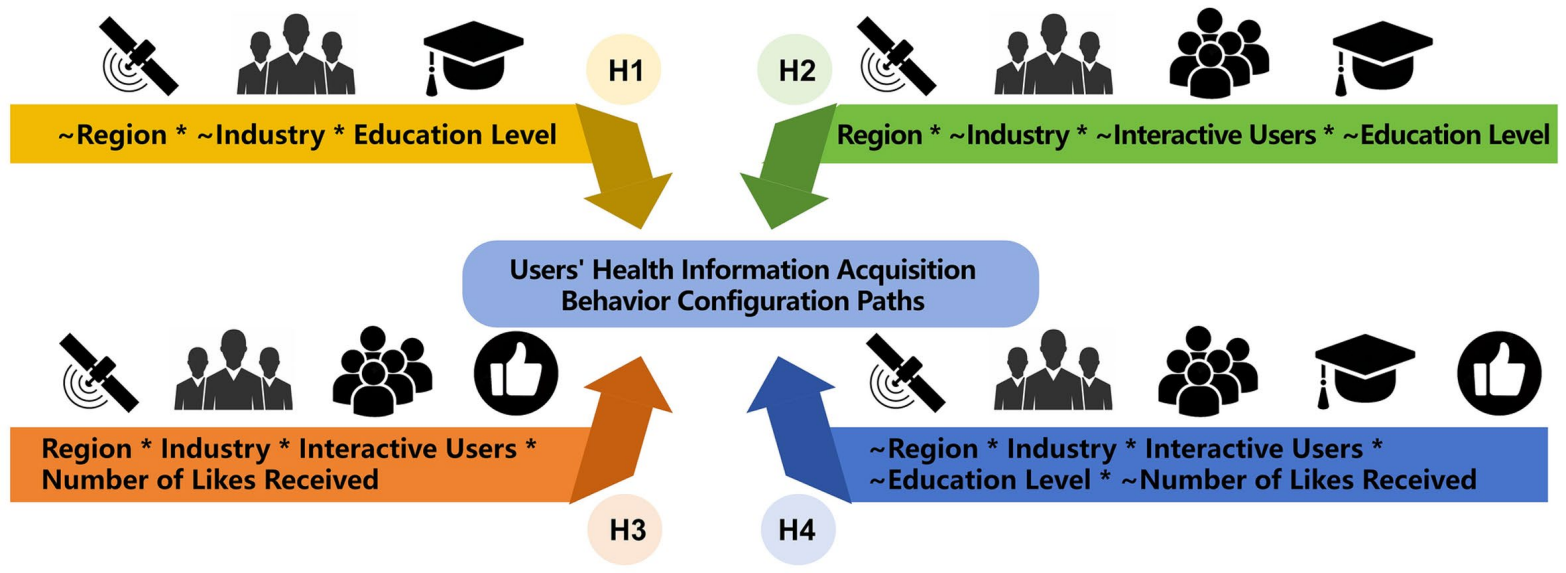
Hot topic health information configuration paths.

Based on the analysis of the impact of single antecedent variables and the data analysis results, it is evident that the impact of single factors on users’ acquisition behavior of health information is not significant and not a sufficient necessary condition. Therefore, this study further explores the configurational effects among the antecedent variables, ultimately resulting in the identification of four configurational paths, as shown in Figure 2

1 H1: ~*Region* * ~ *Industry* * *Education Level*.

Configuration one signifies a scenario where the local and industry contexts do not significantly impact users’ acquisition of health information. Users with higher education and a professional background related to health can more readily access health information. Specifically, highly educated individuals typically possess robust health information screening abilities and a high level of health awareness. If the user’s professional background is directly related to health, they are more likely to obtain targeted health information. For instance, groups with strong academic backgrounds such as medical students, public health scholars, and nutritionists can directly access relevant health information from professional literature, academic resources, or industry news. Therefore, in this context, education level becomes a key driving factor in health information acquisition. Even in the absence of specific regional advantages or industry relevance, users’ high levels of cognitive capital can still facilitate their effective acquisition of health information.

This pathway reflects the predominant role of cognitive social capital (education, professional background) in health information acquisition. Users with higher education and a professional background related to health can, through their precise screening abilities, actively engage with high-quality health information, enhancing both the efficiency and effectiveness of their health information acquisition.

2 H2: *Region* * ~ *Industry* * ~ *Interactive Users* * ~ *Education Level*.

Configuration two indicates that users in economically developed regions can still access health information effectively, even when the industry is unrelated, there is limited interaction among users, and education levels are not high. In regions with advanced economic development, the prevalence of the internet and mobile devices is high, and residents have stronger network access and information circulation capabilities. Therefore, even if users’ industries are not related to health or their social network interactions are sparse, as long as they are in these resource-rich regions, they can access a wealth of health information through online platforms, social media, and other channels.

This pathway underscores the driving role of regional disparities in structural social capital in health information acquisition. The infrastructure, prevalence of information technology, and accessibility of online platforms in economically developed regions enable users to effectively access health information without other conditions. Consequently, geographical location and the level of regional economic development will directly affect the channels and resources available to users for obtaining health information.

3 H3: Region * Industry * Interactive Users * Number of Likes Received.

Configuration three suggests that when users are located in economically developed regions, engaged in health-related industries, and possess abundant relational and cognitive social capital (such as active interaction users and a high number of likes), they can access a broader range of health information. This pathway emphasizes the synergistic effects of multidimensional social capital. Specifically, the combination of region and industry provides users with high-quality information sources and industry-specific health knowledge; active interaction users and the number of likes represent the level of engagement of the user on social platforms and directly impact the dissemination and acceptance of health information. An active network of interactions facilitates faster and wider information flow, while a high number of likes signifies greater recognition and dissemination of information, thereby enhancing its credibility and effectiveness.

This configuration pathway reveals the interactive effects of structural, relational, and cognitive social capital. In economically developed regions, professionals in health-related industries can expand the breadth and depth of information acquisition through social interactions and information sharing. Furthermore, through the accumulation of ongoing interaction relationships (such as fans, followers) and the social recognition they receive (such as the number of likes), the dissemination of health information can be accelerated, making information acquisition more efficient and diverse, highlighting the significant role of social networks and social capital in health information acquisition behaviors.

4 H4: ~*Region* * *Industry* * *Interactive Users* * ~ *Education Level* * ~ *Number of Likes Received*.

Configuration four indicates that users in regions with underdeveloped economies and a lack of cognitive social capital can still obtain health information by engaging in high-frequency online community interactions and working in health-related industries. In regions with adverse economic conditions, users can compensate for other deficiencies by increasing the frequency of social interactions (such as participating in health-related forums) and choosing to work in health-related industries, thereby enhancing the efficiency and effectiveness of information acquisition.

This pathway reflects how individuals can overcome shortcomings in structural social capital (region, industry) and cognitive social capital (education, number of likes) through self-regulation and optimization of their social capital. This path demonstrates the flexibility and adaptability of individuals, illustrating their strategic construction and utilization of social capital to improve the ability to search for health information in environments where information and resources are relatively scarce. In summary, for the acquisition of health information, users do not need to possess all three types of social capital—structural, relational, and cognitive—but instead, leverage their enriched social capital while making the most of their strengths and minimizing their weaknesses to acquire health information.

## 6 Conclusion

### 6.1 Summary

With the development of the internet, social Q&A platforms have become important channels for users to access health information. However, there are significant differences in health information access behavior among users, which are closely related to variations in their social capital. This study, based on health information case examples from social Q&A platforms, utilizes social capital theory and fuzzy set qualitative comparative analysis (fsQCA) to explore the multidimensional factors influencing user health information access behavior and their interactions. The study identifies four configuration paths of health information access behaviors and reveals how structural, relational, and cognitive social capital impact the process of health information acquisition.

The findings indicate that structural social capital plays a significant role in users’ health information access. Specifically, the economic development level of the user’s region directly affects the availability of technological devices and resources, which in turn enhances information literacy. In economically developed regions, users not only have access to better technological equipment but are also more capable of effectively utilizing the internet to access health information. The enhancement of information literacy enables users to actively engage in various “capital enhancement” activities in the online environment, thereby expanding their channels for accessing health information (50). Furthermore, the study reveals that even in the absence of one dimension of social capital, users can compensate for the deficiency by strengthening other dimensions, thereby optimizing their health information access paths. For example, users can enhance their relational social capital by increasing online interaction frequency, or they can improve their cognitive social capital through continuing education, thus compensating for the deficiencies in other dimensions.

Therefore, the study concludes that the disparities in health information access are not only related to the overall level of social capital but also closely linked to the interaction and combination of different dimensions of social capital. This finding provides a new perspective for health information dissemination. In the digital information era, access to health information is not merely a reflection of individual information literacy but also the result of accumulated social capital. The public can optimize their health information access paths by enhancing certain dimensions of social capital to compensate for deficiencies in others. This finding offers theoretical support for public health promotion and health information dissemination strategies. Future research could further explore how enhancing specific dimensions of social capital can promote broader access to and utilization of health information.

### 6.2 Limitations of the study

While this study provides some theoretical references for optimizing the allocation of health resources and enhancing the efficiency of users’ health information acquisition behavior through the study of configurational pathways influencing such behaviors, it still has several limitations.

Firstly, the study is based on a sample of Q&A cases within the “Health” topic on the Zhihu social Q&A platform. Although Zhihu has a large user base and high activity levels in China, selecting a single platform as the research sample introduces selection bias. Part of this bias stems from the particularity of Zhihu’s user population, which is predominantly composed of young, highly educated individuals with a strong interest in health topics, thus the findings may not fully represent the health information acquisition behaviors of all populations. Moreover, as a social Q&A platform, Zhihu’s user interaction and information sharing characteristics differ from those of other social platforms (such as Weibo, TikTok, Facebook, etc.), which limits the generalizability of the research.

Secondly, in terms of variable selection, the article has not comprehensively considered all factors that may influence users’ health information acquisition behavior. The selected antecedent variables are primarily based on social capital theory, which provides some theoretical justification but remains somewhat limited. It has not considered other potentially important variables, such as users’ lifestyles, health concepts, and cultural backgrounds. This limitation in variable selection may lead to the omission of some important antecedent variables, thereby affecting the comprehensiveness and explanatory power of the research outcomes. Over time, the user base, interaction patterns, and topic discussion trends on the Zhihu platform may change, and the stability of the conclusions across different time points is worth further verification.

Lastly, considering the significant differences in social platforms, cultural backgrounds, and user behavior habits across different countries and regions, although this study has revealed patterns of health information acquisition in the Chinese context, it remains to be further verified whether these findings are applicable to other countries or regions. In a cross-cultural context, the dimensions of social capital and the methods of health information acquisition may differ. Future research could consider comparing social platforms across different countries or regions as cases to enhance the generalizability of the research outcomes.

## Data Availability

The datasets presented in this study can be found in online repositories. The names of the repository/repositories and accession number(s) can be found in the article/Supplementary material.
