# Supplementary material for: Factors influencing health information acquisition behavior from a social capital theory perspective: a study of Zhihu users
Source: Front Public Health. 2025 Apr 10;13:1501879. doi: 10.3389/fpubh.2025.1501879 (PMC12018415; doi:10.3389/fpubh.2025.1501879)
Supplement: Supplementary file 1 [file Data_Sheet_1.docx]

# Appendix A

Table A1. Direct calibration method process.

| **Topic** | **Comments** | **Likes** | **Total** | **Cross Point**  **Deviation** | **Scalar** | **Product** | **Degree of Membership** |
| --- | --- | --- | --- | --- | --- | --- | --- |
| Is it true that all beverages taste better when served cold? | 47 | 1040 | 1087 | 487 | 0.00125 | 0.60875 | 0.65 |
| Is it true that all beverages taste better when served cold? (2) | 2 | 46 | 48 | -552 | 0.0075 | -4.14 | 0.02 |
| What are the methods for sobering up? | 21 | 428 | 449 | -151 | 0.0075 | -1.1325 | 0.24 |
| What are the methods for sobering up? (2) | 7 | 34 | 41 | -559 | 0.0075 | -4.1925 | 0.01 |
| A woman suffered from facial paralysis after riding a bicycle with wet hair. | 33 | 273 | 306 | -294 | 0.0075 | -2.205 | 0.10 |
| A woman suffered from facial paralysis after riding a bicycle with wet hair. (2) | 8 | 7 | 15 | -585 | 0.0075 | -4.3875 | 0.01 |
| What fruits are best for people with diabetes to consume? | 39 | 114 | 153 | -447 | 0.0075 | -3.3525 | 0.03 |
| Are there any benefits to being overweight? | 206 | 3321 | 3527 | 2927 | 0.00125 | 3.65875 | 0.97 |
| Are there any benefits to being overweight? (2) | 735 | 735 | 1470 | 870 | 0.00125 | 1.0875 | 0.75 |
| A 16-year-old boy died after choking on a pearl milk tea, his family claims it was due to sudden cardiac arrest. How should one handle such situations promptly? | 82 | 504 | 586 | -14 | 0.0075 | -0.105 | 0.47 |
| How can men maintain their health daily? | 28 | 558 | 586 | -14 | 0.0075 | -0.105 | 0.47 |
| Could you recommend some toothpaste that truly has whitening effects on teeth? | 60 | 295 | 355 | -245 | 0.0075 | -1.8375 | 0.14 |
| Could you recommend some toothpaste that truly has whitening effects on teeth? (2) | 15 | 163 | 178 | -422 | 0.0075 | -3.165 | 0.04 |
| Do foreign girls drink ice water and their mothers don't tell them it's bad for their health and may cause cold in the womb? | 915 | 2374 | 3289 | 2689 | 0.00125 | 3.36125 | 0.97 |
| What signs on your body indicate that you are aging? | 66 | 710 | 776 | 176 | 0.00125 | 0.22 | 0.55 |
| What is your view on the article published in "Science" that suggests middle-aged weight gain is not due to a slowing metabolism? | 233 | 2376 | 2609 | 2009 | 0.00125 | 2.51125 | 0.92 |
| What is your view on the article published in "Science" that suggests middle-aged weight gain is not due to a slowing metabolism? (2) | 25 | 145 | 170 | -430 | 0.0075 | -3.225 | 0.04 |
| What are the benefits of jogging five kilometers every day? | 486 | 15000 | 15486 | 14886 | 0.00125 | 18.6075 | 1.00 |
| What are the benefits of jogging five kilometers every day? (2) | 699 | 1864 | 2563 | 1963 | 0.00125 | 2.45375 | 0.92 |
| Why is milk still widely promoted in China when most people are lactose intolerant? | 12 | 60 | 72 | -528 | 0.0075 | -3.96 | 0.02 |
| Why is milk still widely promoted in China when most people are lactose intolerant? (2) | 855 | 8817 | 9672 | 9072 | 0.00125 | 11.34 | 1.00 |
| What eating habits do thin people have? | 289 | 721 | 1010 | 410 | 0.00125 | 0.5125 | 0.63 |
| What are your small habits for looking beautiful? | 366 | 9478 | 9844 | 9244 | 0.00125 | 11.555 | 1.00 |
| What does the real life of a gout patient look like? | 110 | 480 | 590 | -10 | 0.0075 | -0.075 | 0.48 |
| Why am I still tired after getting at least eight hours of sleep daily? How can I adjust my body to be energetic? | 43 | 392 | 435 | -165 | 0.0075 | -1.2375 | 0.22 |
| How can one determine which vitamin they are deficient in? | 603 | 32000 | 32603 | 32003 | 0.00125 | 40.00375 | 1.00 |
| Why does a person who brushes their teeth diligently have bad breath? | 32 | 110 | 142 | -458 | 0.0075 | -3.435 | 0.03 |
| How can office workers protect their shoulders and necks from strain? | 1 | 13 | 14 | -586 | 0.0075 | -4.395 | 0.01 |
| How can office workers protect their shoulders and necks from strain? (2) | 145 | 15149 | 15294 | 14694 | 0.00125 | 18.3675 | 1.00 |
| How can one control their uric acid levels to maintain normalcy? | 0 | 15 | 15 | -585 | 0.0075 | -4.3875 | 0.01 |

Table A2. Basic data table.

| **Topic** | **Region** | **Industry** | **Interacting Users** | **Education Level** | **Number of Likes** | **Popularity** |
| --- | --- | --- | --- | --- | --- | --- |
| Is it true that all beverages taste better when served cold? | 1 | 0.67 | 0.67 | 1 | 1 | 0.65 |
| Is it true that all beverages taste better when served cold? (2) | 1 | 0.33 | 0.67 | 0.67 | 0.33 | 0.02 |
| What are the methods for sobering up? | 1 | 0 | 0.67 | 0 | 0.33 | 0.24 |
| What are the methods for sobering up? (2) | 0.67 | 0 | 1 | 0 | 0.33 | 0.01 |
| A woman suffered from facial paralysis after riding a bicycle with wet hair. | 1 | 1 | 0.67 | 1 | 0 | 0.10 |
| A woman suffered from facial paralysis after riding a bicycle with wet hair. (2) | 1 | 0 | 0.67 | 0 | 0.33 | 0.01 |
| What fruits are best for people with diabetes to consume? | 1 | 0 | 0.67 | 0.67 | 0.33 | 0.03 |
| Are there any benefits to being overweight? | 1 | 0.67 | 1 | 0.67 | 1 | 0.97 |
| Are there any benefits to being overweight? (2) | 0.67 | 1 | 1 | 0 | 0.67 | 0.75 |
| A 16-year-old boy died after choking on a pearl milk tea, his family claims it was due to sudden cardiac arrest. How should one handle such situations promptly? | 1 | 1 | 1 | 0 | 1 | 0.47 |
| How can men maintain their health daily? | 0.33 | 0.33 | 0 | 0.67 | 0 | 0.47 |
| Could you recommend some toothpaste that truly has whitening effects on teeth? | 1 | 0 | 0.33 | 0.67 | 0.33 | 0.14 |
| Could you recommend some toothpaste that truly has whitening effects on teeth? (2) | 1 | 1 | 1 | 0.67 | 0.67 | 0.04 |
| Do foreign girls drink ice water and their mothers don't tell them it's bad for their health and may cause cold in the womb? | 1 | 0 | 0.33 | 0 | 0.33 | 0.97 |
| What signs on your body indicate that you are aging? | 1 | 0 | 0.33 | 0.67 | 0 | 0.55 |
| What is your view on the article published in "Science" that suggests middle-aged weight gain is not due to a slowing metabolism? | 1 | 1 | 1 | 1 | 1 | 0.92 |
| What is your view on the article published in "Science" that suggests middle-aged weight gain is not due to a slowing metabolism? (2) | 1 | 0 | 0.67 | 0.67 | 0.33 | 0.04 |
| What are the benefits of jogging five kilometers every day? | 1 | 1 | 1 | 0.67 | 1 | 1.00 |
| What are the benefits of jogging five kilometers every day? (2) | 1 | 0 | 0.33 | 0.33 | 0 | 0.92 |
| Why is milk still widely promoted in China when most people are lactose intolerant? | 1 | 0.67 | 0.67 | 1 | 0.67 | 0.02 |
| Why is milk still widely promoted in China when most people are lactose intolerant? (2) | 0.67 | 0.33 | 1 | 0 | 1 | 1.00 |
| What eating habits do thin people have? | 0.67 | 0 | 0 | 0 | 0.33 | 0.63 |
| What are your small habits for looking beautiful? | 1 | 0 | 0.33 | 0 | 0.33 | 1.00 |
| What does the real life of a gout patient look like? | 0.67 | 1 | 0.67 | 1 | 0.67 | 0.48 |
| Why am I still tired after getting at least eight hours of sleep daily? How can I adjust my body to be energetic? | 1 | 0 | 1 | 0 | 1 | 0.22 |
| How can one determine which vitamin they are deficient in? | 1 | 0.33 | 0.33 | 0.67 | 0.33 | 1.00 |
| Why does a person who brushes their teeth diligently have bad breath? | 0 | 1 | 0.67 | 0 | 0.67 | 0.03 |
| How can office workers protect their shoulders and necks from strain? | 1 | 1 | 0.67 | 0.33 | 0.33 | 0.01 |
| How can office workers protect their shoulders and necks from strain? (2) | 0 | 1 | 0.67 | 0.33 | 0 | 1.00 |
| How can one control their uric acid levels to maintain normalcy? | 0 | 0.67 | 0 | 0 | 0 | 0.01 |
